# Equity and Generalizability of Radiomics in Orbital Disease: Challenges for Ophthalmology, Otolaryngology, and Plastic Surgery

**DOI:** 10.3390/diagnostics16070968

**Published:** 2026-03-24

**Authors:** Hana Abbas, Maria Abou Taka, Precious Ochuwa Imokhai, Satyam K. Singh, Christine Gharib, Amaany Mohamed Mehad, Amanda Brooks

**Affiliations:** 1 School of Medicine, Meharry Medical College, Nashville, TN 37208, USA; 2 Department of Medicine, Schulich School of Medicine and Dentistry, London, ON N6A 5C1, Canada; 3 Montana College of Osteopathic Medicine, Rocky Vista University, Billings, MT 59106, USA; 4 College of Medicine, University of Illinois, Chicago, IL 60612, USA; 5 Department of Medicine, California University of Science & Medicine, Colton, CA 92324, USA; 6 College of Medicine, Gulf Medical University, Ajman P.O. Box 4184, United Arab Emirates; 7 Department of Research and Scholarly Activity, Rocky Vista University, Englewood, CO 80112, USA

**Keywords:** radiomics, multi-omics, orbital masses, machine learning, precision medicine, medical imaging, standardized protocols, dataset bias, health disparities, external validation, surgical planning, algorithmic bias, voxel-level analysis, feature extraction

## Abstract

**Background/Objectives**: Radiomics-based machine learning models have demonstrated high accuracy in differentiating benign from malignant orbital masses, with early studies suggesting performance comparable to expert radiologists. However, translation into clinical practice remains limited due to dataset constraints, including retrospective study designs, single-center cohorts, and underrepresentation of diverse patient populations. This review aims to evaluate the current evidence supporting radiomics in orbital disease while critically examining barriers to generalizability and equity across ophthalmology, otolaryngology, and plastic surgery. **Methods**: A narrative literature review was conducted to assess radiomics applications in orbital oncology and reconstruction. Studies evaluating diagnostic accuracy, margin assessment, postoperative surveillance, and surgical planning across ophthalmology, head and neck surgery, and reconstructive surgery were analyzed, with particular attention paid to dataset composition, validation strategies, and imaging standardization. **Results**: Radiomics models demonstrated high diagnostic performance in differentiating orbital tumors, optimizing surgical planning, and aiding postoperative monitoring. However, most studies relied on small, homogeneous datasets lacking racial, ethnic, and pediatric representation. External validation was uncommon, and imaging heterogeneity limited reproducibility. These deficiencies restrict the clinical translation of radiomics and risk exacerbating healthcare disparities, particularly among underrepresented populations. **Conclusions**: Radiomics holds promise as a precision medicine tool for orbital diagnosis, surgical navigation, and postoperative care. Nevertheless, its clinical adoption is constrained by dataset bias, lack of standardization, and limited prospective validation. Future progress requires multi-institutional, demographically diverse datasets and standardized imaging protocols to ensure equitable and generalizable implementation across specialties.

## 1. Introduction

Radiomics, defined as the high-throughput methodology for converting routine medical images (computerized tomography (CT)/magnetic resonance imaging (MRI)/positron emission tomography (PET)) into quantitative, mineable features, is increasingly being modeled to improve diagnostic accuracy, risk stratification, and treatment planning in oncology [1,2,3]. Since clinical assessment in orbital disease is often restricted by deep location and indistinct phenotypes, standard imaging remains fundamental to the work-up; radiomics supplements this process by extracting subtle, subvisual patterns potentially linked directly to histopathology and underlying tumor biology [4,5]. The orbit poses a unique surgical challenge due to its compact, cone-shaped confines, which tightly pack the globe, extraocular muscles, lacrimal gland, optic nerve, and vascular bundles adjacent to the skull base and paranasal sinuses; consequently, patient care is inherently multidisciplinary, involving ophthalmology, ENT, neurosurgery, and plastic surgery, where surgical corridors must be meticulously planned [4]. Initial orbital radiomics research suggests utility in distinguishing entities with similar appearances, such as ocular adnexal lymphoma from idiopathic orbital inflammation, and characterizing benign tumors, which could guide surveillance protocols and operative strategies [6,7,8]. Moreover, in adjacent anatomical regions like the head-and-neck and skull-base, quantitative texture and shape signatures have successfully aided tumor grading and type discrimination, hinting at valuable future applications for margin assessment and surgical approach selection in orbit-adjacent malignancies [9,10]. Clinical translation remains challenging because most published orbital radiomics studies rely on retrospective, single-center cohorts of modest size, leading to limited generalizability [2,11]. Critical issues include a scarcity of external validation, poor representation of multi-ethnic patient populations, and active concerns regarding methodological standardization and feature reproducibility, even with guidance from initiatives like the Image Biomarker Standardization Initiative (IBSI) [11,12]. These combined deficits—encompassing dataset bias, lack of validation, and underrepresentation—form the primary motivation for this review focused on advancing radiomics in orbital oncology and reconstruction [1].

### Radiomics in Orbital Oncology: Current Evidence and Limitations

Current evidence concentrates on diagnostic differentiation, most notably distinguishing ocular adnexal lymphoma (OAL) from idiopathic orbital inflammation (IOI). Deep-learning models integrating multimodal radiomic features with clinical variables have reported area-under-the-curve (AUC) values approaching 0.95 for distinguishing OAL from IOI, reflecting excellent discrimination and implying a high probability that the model assigns higher predicted probabilities to true lymphoma cases than to inflammatory cases [6]. However, many studies do not report confidence intervals, clinically actionable sensitivity–specificity thresholds, or prospective validation. Consequently, it remains uncertain whether these models would meaningfully alter clinical decision-making, such as guiding biopsy decisions or treatment selection. Similarly, an MRI-based radiomics nomogram supported individualized preoperative probability estimation in an independent test set for OAL versus IOI differentiation [6]. For benign orbital tumors, MRI-radiomics successfully distinguished cavernous hemangioma (OCH) from schwannoma (OSC) with accuracies around 90+%, suggesting utility where conventional imaging sequences present overlapping findings [8]. Although direct, head-to-head comparisons with expert readers are limited in the orbital setting, baseline radiologist performance shows variable balanced accuracy across common orbital neoplastic categories (e.g., 0.82–0.85 for neoplasm), highlighting room for quantitative tools to augment human interpretation rather than replacing it outright [5,13]. Clinically, more accurate preoperative labeling of lymphoma versus inflammation could significantly streamline treatment paths, guiding biopsy decisions, radiotherapy planning, and corticosteroid trials [8]. Furthermore, refined benign-tumor subtyping aids in refining surveillance intervals and optimizing surgical approach selection, particularly in anticipating the neurovascular planes surrounding cavernous hemangioma [4].

Limitations recur uniformly across existing orbital radiomics studies. Most underlying datasets are retrospective, single-institution cohorts sourced from limited geographic regions with modest patient numbers (e.g., OAL/IOI series of approximately 90–100 patients), generating concerns regarding spectrum bias and external generalizability [6]. Crucially, external validation is uncommon or relies on excessively small hold-out sets, truly prospective studies are essentially absent, and few published works assess essential clinical impact endpoints such as changes in management, successful biopsy yield, margin negativity, or improved vision-related outcomes [6]. The implementation pipelines vary widely, encompassing disparate parameters such as segmentation scope (from the lesion only to the “whole-orbit”), diverse feature extraction software, and non-standardized preprocessing techniques, collectively threatening reproducibility and transportability across institutions [3,11,14]. Finally, while analogous radiomics studies focusing on adjacent skull-base and brain tumors demonstrate the feasibility of predicting tumor grade or type—information crucial for surgical margin and corridor planning—orbit-specific prospective trials explicitly linking radiomics data to critical operative decisions and outcomes remain a significant unmet need [9,10,11].

## 2. Materials and Methods

### 2.1. Study Design

This study was conducted as a narrative literature review examining the current evidence supporting radiomics applications in orbital disease across ophthalmology, otolaryngology–head and neck surgery, and plastic and reconstructive surgery. The review focused on diagnostic modeling, preoperative margin assessment, postoperative surveillance, periorbital invasion prediction, and reconstructive vascular imaging, with particular attention to issues of dataset bias, generalizability, and equity.

This study was conducted as a narrative literature review rather than a systematic review or meta-analysis. Accordingly, the search strategy was designed to identify representative studies and emerging themes in orbital radiomics rather than exhaustively capture all eligible publications. Formal systematic-review procedures such as duplicate screening, protocol registration, and quantitative risk-of-bias scoring were therefore not performed.

### 2.2. Literature Search Strategy

A structured search of PubMed/MEDLINE, Embase, and Google Scholar was performed to identify relevant articles published between January 2010 and January 2026. The search strategy combined controlled vocabulary and free-text terms related to:

- “radiomics”;
- “orbital tumors”;
- “ocular adnexal lymphoma”;
- “idiopathic orbital inflammation”;
- “sinonasal tumors”;
- “skull base”;
- “periorbital invasion”;
- “machine learning”;
- “margin assessment”;
- “postoperative surveillance”;
- “flap monitoring”;
- “reconstructive surgery imaging”;
- “dataset bias”;
- “health disparities”;
- “pediatric imaging”.

Reference lists of eligible studies were manually screened to identify additional relevant publications.

### 2.3. Study Selection

Studies were included if they:

1. Evaluated radiomics or machine learning-based quantitative imaging in orbital disease or adjacent skull base/sinonasal pathology with orbital involvement;
2. Reported diagnostic performance metrics (e.g., AUC, sensitivity, specificity), predictive modeling, or clinical application in surgical planning or postoperative monitoring;
3. Discussed imaging protocol considerations, segmentation strategies, validation techniques, or reproducibility;
4. Addressed demographic composition, external validation, or equity-related concerns.

Studies focusing solely on conventional imaging without radiomic feature extraction were excluded unless they provided important contextual comparison data. After full-text review, 120 studies met the inclusion criteria and were included in the final qualitative synthesis.

Consistent with the narrative-review methodology, formal systematic-review risk-of-bias scoring tools (e.g., ROBIS or QUADAS-2) were not applied; instead, methodological limitations such as retrospective design, small sample size, and lack of external validation were qualitatively assessed.

## 3. Current Evidence and Technical Limitations

### 3.1. Preoperative Margin Assessment in Orbital Tumors

This section summarizes key findings from the literature while highlighting methodological limitations and translational gaps identified across studies. Although several radiomics models have been developed for orbital tumors, few directly address preoperative margin assessment, illustrating a critical gap in enhancing surgical precision in ophthalmologic oncology. Present reports focused on higher-order diagnostic applications, including differentiating benign from malignant lesions or segmenting large anatomical structures like the optic nerve or extraocular muscles, rather than analyzing microscopic tumor margins [15,16]. Additionally, most of the published models that investigated macroscopic applications relied on high-level imaging features that were extracted from MRI or CT scans to identify common orbital diseases, such as cavernous hemangiomas, schwannomas, and lymphomas [17,18].

Despite progress in orbital radiomics, particularly in macroscopic applications, a noticeable gap remains in addressing subclinical or microscopic margin detection. Accurate microscopic margin detection is crucial for surgical planning as it determines the precise boundaries between healthy and diseased tissue associated with the orbit [19,20]. In turn, this can influence decisions on the extent of tissue removal and preservation of critical ocular structures during presurgical preparation [21]. To enhance preoperative margin assessment in orbital oncology, radiomics may benefit from improved predictive granularity by quantifying image-derived tumor characteristics, such as nonlinear tumor boundaries, at the voxel level [22]. In turn, surgeons would be able to preserve optic nerve margins, which is critical to minimizing post-operative vision loss.

For instance, while Chen et al. (2021) developed MRI-based radiomics models with nearly perfect diagnostic performance (AUC ≈ 0.98) in differentiating orbital cavernous hemangiomas from orbital schwannomas, this accuracy only applies to broad tumor classification [8]. In particular, these models did not provide spatial data on tumor margin status or subclinical invasion, which are critical for surgical guidance. Similarly, Shao et al. (2023) used T1- and T2-weighted imaging MRI sequences to distinguish IgG4-related orbital disease (IgG4-ROD) from mucosa-associated lymphoid tissue lymphoma (MALT) in a 50-patient cohort (20 IgG4-ROD, 30 MALT) [23]. Their SVM classifiers achieved AUCs between 0.72 and 0.82, indicating that conventional MRI texture features can aid in diagnostic differentiation, though they remain coarse-level tools that are insufficient for margin-specific guidance.

Furthermore, advanced imaging biomarkers can offer indirect physiological proxies for tumor behavior. For example, elevated perfusion or contrast-kinetic parameters indicate neovascularity and leaky vasculature beyond visible tumor boundaries [24,25]. In neuro-oncology and head and neck cancers, integration of these quantitative biomarkers with radiomic analysis enabled the prediction of peritumoral infiltration and margin status [26,27]. However, these integrative frameworks are currently absent in orbital oncology. To advance this field, orbital radiomics pipelines need to adopt similar approaches by co-registering physiological imaging parameters with histopathological margin data and defining segmentation targets beyond the visible tumor borders.

### 3.2. Postoperative Surveillance (Recurrence Detection)

While postoperative recurrence is a major challenge in orbital oncology, no published study to date applied radiomics-based surveillance for residual or recurrent disease in the orbital region. To illustrate, Tang et al. (2013) retrospectively studied 253 patients with recurrent orbital space-occupying lesions [28]. Through clinicopathological correlation, they recorded recurrence intervals, categorized lesion-type distributions, and analyzed histopathological subtypes. However, this study did not integrate advanced imaging, nor did it model margin status or radiomic predictors of recurrence. As such, these findings underscore the clinical burden of recurrence, while simultaneously demonstrating a critical translational gap in orbital-oncology imaging research.

In other oncology studies, radiomics was successfully used to predict recurrence and survival endpoints of disease. For instance, Aerts et al. (2014) demonstrated a CT-based radiomic signature that predicted overall survival and local control in patients with lung and head and neck cancers [29]. Additionally, Liao et al. (2020) developed MRI-based models for recurrence-free survival for patients with nasopharyngeal carcinoma [30]. Together, these studies illustrate how temporal imaging features, such as the quantitative changes in texture or intensity across serial scans, can predict malignant tumor behavior [30,31]. However, there are no analogous frameworks that exist for orbital disease. Without such data, radiomic models remain restricted in their ability to capture subtle temporal changes that precede recurrence, which limits the applications of recurrence prediction in orbital oncology. Furthermore, with the lack of quantitative surveillance, early orbital recurrences may remain difficult to detect prior to symptomatic progression. As such, future efforts should focus on integrating serial MRIs into deep-learning models trained to detect preclinical radiomic shifts suggestive of recurrence.

### 3.3. Limitations with Rare Tumor Types and Pediatric Data

Orbital tumors represent a highly heterogeneous group encompassing both benign and malignant entities, many of which are rare or predominantly occur in children [32,33]. These include rhabdomyosarcoma, optic pathway glioma, and embryonal tumors [34]. Several of these tumors are rare in clinical incidence; for example, orbital rhabdomyosarcoma has an estimated incidence of 0.1–0.3 cases per million children, while optic gliomas represent a small subset of pediatric central nervous system tumors [35]. Despite their low incidence, their limited representation in radiomics research reflects the absence of sufficiently annotated imaging datasets rather than epidemiological rarity alone. Additionally, the histological diversity of orbital tumors and their proximity to critical neurovascular structures further complicate the development of robust radiomic models. Consequently, most published orbital radiomics studies focus on adult patient cohorts with common lesions, such as cavernous hemangioma or lymphoma, leaving pediatric and rare tumor variants largely unexplored [36,37]. Therefore, this provides little insight into rarer and/or pediatric pathologies where differences in imaging and biological behavior are substantial.

Notably, in pediatric populations, ongoing craniofacial growth, smaller orbital volumes, and variable tissue composition introduce challenges for radiomic reproducibility [38,39]. In turn, these changes in bone and soft tissue architecture exhibited in pediatric populations alter spatial reference points used for serial comparisons, thereby affecting the consistency of imaging features and complicating the alignment of serial scans over time [40,41]. Pediatric patients also often exhibit motion artifacts from limited cooperation during imaging sessions and differing scanner protocols across the country, which lead to impaired segmentation consistency and feature stability across serial time points [42]. Moreover, demographic reporting within existing pediatric datasets is often incomplete. Specifically, there are few reports that stratify study findings by age, race, or histological subtype, which limits both external study validity and equity in model performance across the diverse pediatric patient groups located in the United States [43,44].

Overall, radiomics frameworks trained on adult or common tumor datasets may not generalize well to pediatric populations or rare tumor variants. Without multicenter repositories incorporating adequate pediatric representation and standardized imaging protocols, radiomics will remain clinically limited for margin-level assessment or recurrence prediction in orbital oncology. Addressing these gaps through pediatric-specific radiomics pipelines and diverse training datasets is essential for equitable clinical translation.

### 3.4. Otolaryngology—Head and Neck Surgery—Sinonasal/Skull Base Tumors with Orbital Invasion

In advanced cases, sinonasal and anterior skull base cancers, such as sinonasal squamous cell carcinoma (SCC) and inverted papilloma (IP), invade the orbit, disrupting surgical boundaries and jeopardizing oncological safety and patient functional outcomes [45,46]. Specifically, these cancers impact the orbit in three primary ways: they erode the lamina papyracea, spread along the infraorbital and ethmoidal nerves and vessels, or directly extend through the medial orbital wall [47,48]. As such, accurate imaging, particularly through advanced radiomics, is vital for surgical planning. Once invasion exceeds the periorbita or involves extraocular muscles, the balance between oncologic clearance and globe preservation becomes a key determinant of patient morbidity and their quality of life.

The use of radiomics in head and neck oncology cases are currently more mature than those in orbital oncology, especially in modeling tumor invasion patterns. For instance, in head and neck cancers, radiomics models were developed to quantify tumor heterogeneity and to predict aggressive tumor behavior using multiparametric MRI and CT datasets [27,45]. In turn, these predictive frameworks generate quantitative imaging signatures detailing tumor aggressiveness, local invasion, and patient survival outcomes, which may subsequently inform treatment planning and surgical decision-making [27,45]. Although these models were originally developed for head and neck oncology, their methodological frameworks may be adaptable to orbital oncology. For example, texture-based features and spatial heterogeneity metrics used for characterizing skull base and head and neck tumors could be applied to orbital boundaries such as the periorbital fat interface or the lamina papyracea [45]. As such, by adapting these established modeling approaches, orbital radiomics may benefit from methodological advances that are already validated in sinonasal and skull base tumors.

Understanding the extent of orbital invasion requires a radiomics perspective, particularly at the boundary where sinonasal disease meets orbital fat or musculature. This approach provides detailed insights into the microscopic level of invasion, offering a more nuanced understanding than conventional imaging. Unlike conventional imaging, which lacks cellular-level resolution, radiomics provides quantitative signals that may indicate subclinical periorbital invasion [49,50]. Moreover, the limitations of histopathological correlation, which is typically restricted to tissue samples collected during surgery, highlight the need for advanced imaging techniques like radiomics [50]. As such, developing radiomic signatures to measure differences in texture or blood flow across the medial orbital wall might help detect early signs of tumor invasion and assist in choosing the correct surgical approach.

### 3.5. Use of Radiomics for Predicting Periorbital Spread

Recent advances in radiomics and radiogenomics have demonstrated potential in predicting local tumor extension patterns, including periorbital spread in sinonasal malignancies, building on the need for detailed imaging insights. For example, Xia et al. (2024) developed a multiparametric MRI-based radiomics nomogram integrating T1-, T2-, and contrast-enhanced sequences to predict malignant transformation in sinonasal IP [51]. Trained on 146 patients and externally validated, this model achieved an AUC of 0.92 and outperformed conventional radiologic assessment [51]. Notably, the most predictive features arose at the tumor-periorbital interface, including the gray-level co-occurrence matrix entropy, which may suggest that textural disruption at boundary zones could correspond to early microscopic tumor invasion [51].

Furthermore, Yan et al. (2022) achieved a comparable AUC (0.89) in predicting IP malignant transformation across multiple hospital centers, identifying tumor shape irregularity and high-frequency texture as dominant predictors [52]. Importantly, these predictive features cluster near the lamina papyracea and orbital floor, areas commonly affected by early tumor invasion [52,53]. Additionally, Gu and colleagues (2022) built on these findings with an MRI radiomic tool that distinguishes between benign and malignant sinonasal tumors, achieving an AUC of 0.87 [54]. In this study, they retrospectively applied the tool to lesions near the medial orbital wall, highlighting its limitations due to consistent false positives in cases of histologically confirmed subperiosteal invasion [54]. In turn, this raises concerns about unnecessary orbital wall resections or overtreatment in borderline cases, underscoring the need for refinement in radiomic tools. Furthermore, Wang et al. (2025) applied contrast-enhanced CT radiomics to predict IDH1 mutation status, a genomic marker associated with aggressive tumor behavior and local invasion in head and neck SCC, achieving an AUC of 0.90 [55]. Overall, Wang et al. (2025) suggest that vascular-texture features may act as a genomic surrogate of orbital infiltration risk, potentially informing preoperative surgical margins [55].

### 3.6. Impact on Surgical Planning, Endoscopic vs. Open Approaches

Importantly, these advances have direct implications for surgical planning in sinonasal and skull base oncology. In particular, radiomics-based prediction of orbital invasion provides immediate insights that influence surgical strategy, especially in selecting between endoscopic and open craniofacial approaches [56]. To illustrate, Qi et al. (2023) demonstrated that radiomic signatures from contrast-enhanced CT scans and MRI could stratify head and neck tumors by local invasiveness, identifying imaging phenotypes associated with bone erosion, dural abutment, and orbital extension [57]. As a result, Qi et al. (2023) suggest that texture heterogeneity and gradient-based metrics can serve as preoperative markers to help surgeons determine whether endoscopic resection with medial wall preservation is feasible or if open craniofacial access is necessary [57].

Furthermore, He et al. (2024) applied radiomics to differentiate IP from malignant sinonasal neoplasms, reporting AUC values exceeding 0.90 [58]. Their model identified subtle changes in tissue structure and texture near the lamina papyracea, which was linked to early orbital invasion [58,59]. Overall, this model demonstrates the way in which surgeons can make precise decisions by actively using imaging, such as opting for selective decompression rather than extensive surgery.

Together, these studies revealed the evolving role of radiomics in personalized skull base surgery, while highlighting the potential of radiomics to offer a data-driven framework that can inform surgical planning by balancing oncological safety with functional preservation.

### 3.7. Plastic and Reconstructive Surgery—Flap Selection and Vascular Mapping

In recent years, advancements in vascular imaging, including the FlapMap visual language system and 3D-printed vascular models, significantly influenced the planning and execution of complex flap procedures in reconstructive surgery [60,61]. These technologies provide detailed vascular maps that allow for precise flap design, optimizing perfusion while minimizing donor-site morbidity [62]. Traditional tools, such as handheld Doppler or static CTA overlays, limit spatial context, whereas the FlapMap visual language system generates standardized, color-coded maps of perfusion territories [63,64]. In turn, the FlapMap visual language system enhances communication between radiologists and surgeons regarding inflow and drainage patterns, which is crucial for surgical planning in areas like head and neck reconstructions where vascular anatomy is highly variable.

Three-dimensional printed vascular models (3DVMs), which are created from CT angiography data, replicate each patient’s perforator course and branching pattern, allowing surgeons to practice and refine their preoperative approach to flap elevation [65,66]. Furthermore, studies show that using 3DVMs significantly diminishes harvest time and enhances surgical confidence, particularly in procedures involving flaps such as the deep inferior epigastric perforator (DIEP) or anterolateral thigh (ALT) [67,68]. These printed models provide tactile and spatial feedback, bridging the gap between two-dimensional scans and intraoperative anatomy.

Additionally, magnetic resonance angiography (MRA) has become increasingly accepted as a contrast-free, radiation-free alternative to CTA of perforator anatomy, making it especially valuable for patients with renal impairment or those needing repeat imaging [69,70]. In particular, de Geer et al. (2025) introduced an MRA-based workflow integrating high-resolution angiography with personalized 3D-printed models for head and neck reconstruction [71]. Their model demonstrated accurate visualization of submillimeter perforators and venous outflow pathways, enabling individualized flap design and reducing intraoperative uncertainty [71]. As such, this approach highlights MRA’s ability to generate detailed, patient-specific vascular roadmaps without ionizing radiation, paving the way for broader non-contrast imaging adoption in reconstructive microsurgery.

Finally, optical imaging methods, like photoacoustic tomography (PreFlap), are emerging as real-time intraoperative tools. By combining optical contrast with ultrasound resolution, PreFlap visualizes subdermal vessels and microvascular flow before incision [72,73]. In turn, this reduces the risk of ischemia and unnecessary dissection during head and neck reconstruction [74]. Importantly, these technologies not only enhance current surgical planning and execution but also suggest a future where imaging and radiomics could further personalize flap design by quantifying vessel density, tortuosity, and flow patterns.

Despite the potential of these innovations to enhance surgical outcomes, their broad implementation is hindered by the lack of extensive clinical validation, which is necessary to confirm their efficacy and safety. This highlights a trend in data-driven vascular mapping that may enhance surgical planning and patient outcomes.

### 3.8. Monitoring Flap Viability with Imaging Biomarkers

Quantitative imaging biomarkers detect perfusion deficits before visible clinical signs appear. These tools provide surgeons with a critical window to intervene while a flap is still salvageable. For instance, Ota et al. (2022) demonstrated the critical predictive value of CT and MR perfusion imaging in 166 head and neck reconstructions [75]. Perfusion parameters, such as blood flow and mean transit time, which surgeons measured 2 to 4 days after surgery, allowed them to intervene early by identifying flaps at risk of ischemia before necrosis developed, significantly improving salvage rates [75]. Moreover, Razavi et al. (2022) took this a step further by using serial imaging to track volume loss in non-osseous free flaps in patients with a history of oral cavity and/or oropharyngeal cancers [76]. They found that even subtle reductions in flap volume during the first postoperative week correlated with later fibrosis and contour loss [76]. Overall, these findings demonstrate how early volume shifts presage downstream contour deformities, including fibrosis and functional asymmetry, such as facial hollowing or nasolabial collapse [76,77].

In a larger prospective study, Hennocq et al. (2025) analyzed nearly 500 CT and 150 MRI scans from 166 osseous flaps, including fibula, DCIA, and scapular flaps [78]. Specifically, they quantified how flap volume changed over time and they proposed practical overcorrection thresholds for each donor site to counteract predictable resorption, thereby providing surgeons with evidence-based guidance for preoperative design [78].

Moreover, non-ionizing imaging tools, like hyperspectral imaging (HSI), are emerging as valuable bedside monitors [79]. To illustrate, Thiem et al. (2021) showed that HSI detected perfusion failure four to five hours earlier than typical clinical assessment, while Felicio-Bregel et al. (2024) validated its reliability for intraoral free flaps, demonstrating consistent oxygenation readings across serial measurements [80,81]. HSI thus provides a non-contact, real-time map of hemoglobin oxygenation that complements clinical inspection.

In summary, these studies demonstrate that perfusion, volume, and spectral biomarkers can assist surgeons in identifying at-risk flaps sooner, effectively tailoring interventions, and predicting reconstructive outcomes with greater accuracy and safety.

### 3.9. Challenges with Heterogeneity in Imaging Protocols

While imaging biomarkers offer significant advancements, inconsistent imaging protocols remain a major barrier to their extensive implementation. Despite these advances, variability in scanner technology, acquisition timing, and post-processing continues to impede reproducibility across hospital centers [82,83]. For example, Kim et al. (2017) found that postoperative CT indicators of delayed flap failure, such as gas, fluid, or loss of enhancement, varied depending on when and how scans were performed, highlighting the need for standardized scanning protocols and timing guidelines [84,85].

To ensure the broad implementation of standardized imaging protocols, equitable access to advanced imaging technologies is imperative, as they are predominantly concentrated in tertiary care centers [85,86]. This limits the development of diverse, generalizable datasets necessary for training robust predictive models, especially in underserved regions. Without standardized imaging and shared repositories, radiomics models risk being accurate only within a single institution or demographic group.

Overall, addressing these challenges will require multicenter collaboration and the establishment of international working groups to develop consensus guidelines for the integration of radiomics. Importantly, researchers must create shared datasets that involve perfusion, spectral, and volumetric imaging to enable external validation and ensure equitable adoption across various patient care settings. Notably, integrating standardized imaging biomarkers into flap planning and monitoring may transform reconstructive surgery into a precision-guided discipline that may improve overall patient outcomes.

## 4. Discussion

### 4.1. Equity and Generalizability Challenges—Underrepresentation in Current Datasets and Risks of Bias

The application of radiomics in medical research encounters major challenges because of unbalanced datasets which affect both equity and generalizability. The current imaging datasets together with their associated studies show insufficient representation of non-White patients which creates doubts about radiomics model performance in diverse patient groups [87]. The Cancer Imaging Archive (TCIA), which hosts data for radiomics research and training, was found to contain 35–48% fewer Black/African American and Hispanic and American Indian patients than their actual cancer incidence rates in the real world [87]. The authors demonstrated through their research that unbalanced demographics in imaging data reduce the ability of AI tools to work effectively when trained on such datasets, a phenomenon known as dataset shift [88,89,90]. Alarmingly, a systematic review of cancer AI studies revealed that training data racial/ethnic distribution information was absent in 89% of papers but only 4% of studies included this information when developing their models [91]. Patel et al. (2023) identified demographic exclusion as a major problem in medical AI development because algorithms trained without racial considerations will fail to work properly with different patient groups [92]. The lack of diverse patient data in orbital oncology becomes a critical issue because algorithms trained on limited patient groups might fail to detect tumor characteristics that result from genetic or environmental factors which affect diagnostic accuracy and treatment plans for minority groups.

A multitude of different mechanisms may explain why demographic imbalance in imaging datasets reduce the reliability of radiomics models. Biological variability across diverse populations may affect tumor development, including development susceptibility, subtype prevalence, molecular alterations, and growth behaviours, all of which may produce measurable differences in imaging phenotypes captured through radiomic features. Thus, if certain populations are underrepresented in training models these variations may not be adequately represented, limiting model performance when applied to broader patient groups. Technical factors can also contribute to this bias. Differences in scanner types, acquisition parameters, and institutional imaging protocols may introduce systematic variation in imaging features, particularly when datasets are derived from a limited number of institutions or geographic regions [88,89,90].

Another issue arises with the insufficient pediatric data that affects how AI systems perform across different age groups. Most radiomic training datasets lack pediatric orbital tumor cases such as retinoblastoma and orbital rhabdomyosarcoma because these tumors occur rarely in children [92]. A 2025 review discovered that medical imaging data containing children amounts to less than 1% of publicly accessible information although children make up 25% of the worldwide population [93]. The insufficient number of pediatric images in databases results in AI models that function primarily for adult patients which creates problems when used with children. The limited availability of pediatric imaging data leads to both biased predictions and elevated prediction errors when analyzing young patients [93]. For example, a chest X-ray AI system trained on adult data produced prediction errors reaching 50% when analyzing infant patients which demonstrates the dangers of applying this system to children [93]. The development of orbital radiomics models using adult tumors exclusively leads to potential misidentification of pediatric tumors and failure to detect unique imaging features that distinguish pediatric from adult tumors. The research ultimately warns that the insufficient data available for children creates a systematic risk which prevents them from benefiting from AI progress as a result of these limited datasets. The use of single-institution orbital radiomics series creates a problem because models trained on homogeneous adult populations from specific ethnic or regional groups will not perform well in diverse patient groups. Thus, the insufficient representation of non-White, pediatric and international populations in medical models restricts their use in worldwide ophthalmology and oncology practice while creating potential healthcare disparities through biased radiomic evaluation.

### 4.2. Future Directions and Recommendations

The future directions of radiomics research in orbital disease requires precise coordination across multiple specialties to overcome current clinical barriers. Firstly, standardized imaging protocols remain a significant barrier to producing reproducible radiomic features. Multi-center protocols on establishing standardized orbital MRI and CT procedures along with emerging imaging biomarker standards, such as the Image Biomarker Standardization Initiative (IBSI), will be essential for reducing technical variability to facilitate comparisons across several data platforms [94]. The work of IBSI has begun to address this issue by defining consistent feature terminology and reference values to improve reproducibility across institutions and software platforms [12,95]. Similar emerging clinical imaging guidelines for orbital diseases now advocate for standardized MRI and CT acquisition parameters to minimize diagnostic variability. Beyond protocol alignment, quality improvement methods such as phantom calibration and harmonization techniques, including ComBat and GAN-based normalization, are being refined to correct differences across platforms and enhance comparability across multiple datasets [96,97,98]. Thus, creating standardized frameworks through phantom calibration and harmonizing algorithms could allow for multicenter analyses that can improve reproducibility [99]. For example, efforts toward multicenter collaboration are exemplified by large annotated datasets, such as the TOM500 initiative in thyroid eye disease, which demonstrates how shared datasets can enhance the capabilities of radiomic models [99]. As such, shared datasets that integrate clinical, imaging, and histopathologic data across ophthalmology, otolaryngology, and reconstructive surgery fields would enhance generalizability capacities and reduce sampling bias [2,100]. Importantly, including racially and anatomically diverse patient populations, especially from underrepresented areas, will help to prevent algorithmic bias and promote inclusive diagnostic performance.

Currently, most radiomics studies are retrospective and exploratory, which limits future research reproducibility capabilities and highlights the need for designs that recruit patients in advance and use predefined data objectives [101,102]. However, only a small fraction of current orbital radiomics studies have used this design approach [14]. To allow for improved clinical application and standardization, future studies should focus on validating prospective results with standardized methods that use accepted statistical models [103]. For example, standardized imaging protocols defined by IBSI can be incorporated into these study designs [104]. These studies should incorporate longitudinal follow-up to evaluate radiomic stability, outcome prediction, and real-world clinical applicability. Furthermore, integrating imaging and patient treatment outcome data over a longer and clinically significant period of time will also allow researchers to examine the long-term stability of these radiomic features. Recent work using repeated image assessments has demonstrated improved patient risk stratification, emphasizing the clinical importance of using time-dependent models [105,106]. Additionally, the use of radiomics with artificial intelligence (AI)-driven surgical navigation offers a promising option for improving patient outcomes intraoperatively. Preclinical and early clinical studies have already shown that AI-based radiomic models can enhance surgical visualization and planning [107,108,109]. The integration of real-time preoperative radiomic features into augmented realities or surgical navigation systems could assist in delineating tumor margins, optimizing surgical outcomes, and minimizing the risk of iatrogenic injury [110]. Additionally, collaboration between imaging scientists and surgical engineers will be necessary for translating these algorithms into easily usable operative technology that can improve patient outcomes.

Beyond conventional radiomic analysis, integrating imaging features with multi-omic datasets is an important step for improving the biological interpretability of radiomic biomarkers [103,104,105]. In other oncologic fields, previous studies have shown that quantitative radiomic features can be correlated with underlying molecular characteristics. This includes gene-expression, tumor burden, and immune microenvironments. For example, gliomas and head and neck squamous carcinoma have associations reported between specific radiomic patterns and molecular profiles, such as IDH mutations and HPV-related oncogenic pathways [54]. Utilizing a similar approach towards the field of orbital disease could allow for the use of image-derived features to be used as noninvasive indicators of tumor biology. This application is relevant for orbital oncology due to the challenges presented in tissue sampling where there are complex anatomical constraints [107,108,109,110]. Thus, integrating radiomic features with genomic, transcriptomic, and immunologic data can allow for more comprehensive understandings of tumor behavior to improve prognosis, recurrence risk, and therapeutic response. As multi-omic integration fields continue to improve, combined imaging–molecular approaches have the potential to improve clinical precision-medicine strategies.

Ultimately, radiomics may contribute to precision medicine by capturing subtle imaging details that are predictive of molecular behavior and improve patient treatment response. Radiomics allows for noninvasive methods for characterizing tumor microenvironments through MRI, CT, and PET/CT imaging modalities, allowing for better predictions regarding treatment outcomes, disease progression, and overall prognosis across multiple cancer types [111,112]. For example, these radiomic features have been linked to immune cell infiltration, T-cell activity, and molecular subtypes [113]. By linking radiomic biomarkers to genomic, proteomic, and immunologic data, individualized risk stratification and targeted therapeutic planning can be more precisely developed and implemented into patient care [114]. The integration of multi-omics fields will allow for a more comprehensive view of tumor biology and individual patient risk, which could improve predicted patient responses to chemotherapy and immunotherapy, allowing for improved tailored treatment planning [115,116]. In orbital oncology and reconstruction, these integrative approaches have the potential to overcome current challenges regarding personalizing surgical margins, postoperative monitoring, and adjuvant therapy selection by providing image-based biomarkers of disease behavior [117,118]. As studies are already providing promising results demonstrating radiomic and multi-omic combinations, future research may ideally continue to inform risk stratification to further support clinical decision-making and patient outcomes [119].

## 5. Conclusions

Radiomics represents a transformative frontier in orbital oncology and reconstruction, offering the potential to extract quantitative imaging biomarkers that enhance diagnosis, surgical precision, and postoperative surveillance. By translating subtle imaging signals into clinically meaningful data, radiomics can bridge the gap between radiology and pathology, facilitating more accurate preoperative margin assessment, recurrence prediction, and personalized reconstructive planning. However, this promise remains largely unrealized due to fundamental limitations in dataset diversity, standardization, and multicenter validation.

Current orbital radiomics research is constrained by small, homogeneous, and predominantly adult datasets that lack representation from non-White and pediatric populations. This demographic imbalance not only undermines equity but also weakens model generalizability across diverse patient groups, leading to potential diagnostic and therapeutic disparities. Similarly, the absence of standardized imaging protocols, heterogeneous scanner parameters, and limited longitudinal data hinder reproducibility and cross-institutional comparability. These technical and demographic barriers collectively slow the translation of radiomics from proof-of-concept studies into real-world clinical tools. To transition orbital radiomics from experimental modeling to routine clinical application, the following strategic roadmap is proposed:

1. **Global Standardization and Data Inclusivity:** Multicenter initiatives must prioritize the creation of open-access, high-quality repositories that utilize standardized orbital imaging protocols. These datasets should purposefully include pediatric and ethnically diverse cohorts to ensure algorithmic fairness and broad generalizability.
2. **Prospective and External Validation:** Future research must move beyond retrospective pilot studies. The field requires rigorous prospective designs and validation against external, independent cohorts to confirm the reproducibility and reliability of radiomic signatures.
3. **Demonstrable Clinical Integration:** Studies must explicitly link radiomic features to histopathologic findings and longitudinal clinical outcomes. This integration is vital for proving that these models can tangibly improve surgical margin planning, treatment response monitoring, or the prediction of recurrence.
4. **Cross-Disciplinary Synergy:** Developers must work in close coordination with ophthalmologists, otolaryngologists, and reconstructive surgeons. Such interdisciplinary collaboration ensures that computational tools are designed to solve specific, real-world clinical dilemmas rather than existing in a vacuum.

Moving forward, the clinical utility of radiomics will depend on these deliberate collaborations. Unified efforts toward standardized imaging protocols, anchored by initiatives such as the Image Biomarker Standardization Initiative (IBSI), and the creation of multicenter, demographically diverse repositories are essential to achieving equitable and reproducible outcomes. Integrating radiomic data with genomics, proteomics, and immunologic profiles could further advance precision medicine by linking imaging phenotypes to molecular behavior and treatment response.

Ultimately, realizing the full promise of radiomics in orbital disease requires a coordinated global framework that values diversity, transparency, and clinical validation. Only through such collaboration can radiomics evolve from experimental modeling to a clinically trusted tool, transforming orbital oncology and reconstruction into more predictive, personalized, and equitable disciplines.

## Data Availability

No new data were created or analyzed in this study. Data sharing is not applicable to this article.

## Abbreviations

The following abbreviations are used in this manuscript:

AI	Artificial Intelligence
AUC	Area under the curve
CNN	Convolutional neural network
CT	Computed Tomography
CAD	Computer-aided Diagnosis
HNSCC	Head and Neck Squamous Cell Carcinoma
LoG	Laplacian of Gaussian
PACS	Picture Archiving and Communication System
RF	Random Forest
SVM	Support Vector Machine
DICOM	Digital Imaging and Communications in Medicine

## References

[1] Gillies R.J., Kinahan P.E., Hricak H. (2016). Radiomics: Images Are More than Pictures, They Are Data. Radiology.

[2] Lambin P., Leijenaar R.T.H., Deist T.M., Peerlings J., de Jong E.E.C., van Timmeren J., Sanduleanu S., Larue R.T.H.M., Even A.J.G., Jochems A. (2017). Radiomics: The bridge between medical imaging and personalized medicine. Nat. Rev. Clin. Oncol..

[3] van Timmeren J.E., Cester D., Tanadini-Lang S., Alkadhi H., Baessler B. (2020). Radiomics in medical imaging: How-to guide and critical reflection. Insights Imaging.

[4] Vogele D., Sollmann N., Beck A., Haggenmüller B., Schmidt S.A., Schmitz B., Kapapa T., Ozpeynirci Y., Beer M., Kloth C. (2022). Orbital tumors: Clinical, radiologic and histopathologic correlation. Diagnostics.

[5] Lee M.J., Verma R., Hamilton B.E., Pettersson D., Choi D., Kim E.S., Korn B.S., Kikkawa D.O., Rosenbaum J.T. (2024). Utility of orbital imaging in the evaluation of orbital disease. PLoS ONE.

[6] Xie X., Yang L., Zhao F., Wang D., Zhang H., He X., Cao X., Yi H., Hou Y. (2022). Deep learning model combining multimodal radiomics and clinical features for differentiating ocular adnexal lymphoma from idiopathic orbital inflammation. Eur. Radiol..

[7] Yang L., Zhang H., Xie X., Jiang S., Cao X., Hou Y., He X., Wang J., Zhang T., Zhao F. (2023). MRI-based radiomics nomogram for preoperative differentiation between ocular adnexal lymphoma and idiopathic orbital inflammation. J. Magn. Reson. Imaging.

[8] Chen L., Shen Y., Huang X., Li H., Li J., Wei R., Yang W. (2021). MRI-based radiomics for differentiating orbital cavernous hemangioma and schwannoma. Front. Med..

[9] Ko C.C., Zhang Y., Chen J.H., Chang K.T., Chen T.Y., Lim S.W., Wu T.C., Su M.Y. (2021). Preoperative MRI radiomics for prediction of progression and recurrence in meningiomas. Front. Neurol..

[10] Yamazawa E., Takahashi S., Shin M., Tanaka S., Takahashi W., Nakamoto T., Suzuki Y., Takami H., Saito N. (2022). MRI-based radiomics differentiates skull base chordoma and chondrosarcoma. Cancers.

[11] Klontzas M.E. (2024). Radiomics feature reproducibility: The elephant in the room. Eur. J. Radiol..

[12] Whybra P., Zwanenburg A., Andrearczyk V., Schaer R., Apte A.P., Ayotte A., Baheti B., Bakas S., Bettinelli A., Boellaard R. (2024). Image Biomarker Standardization Initiative: Standardized convolutional filters. Radiology.

[13] Lee M.J., Choi D., Kim E.S., Korn B.S., Kikkawa D.O. (2020). Diagnostic accuracy of orbital imaging among radiologists. AJNR Am. J. Neuroradiol..

[14] Zhang Y.P., Zhang X.Y., Cheng Y.T., Li B., Teng X.Z., Zhang J., Lam S., Zhou T., Ma Z.R., Sheng J.B. (2023). Artificial intelligence-driven radiomics study in cancer: The role of feature engineering and modeling. Mil. Med. Res..

[15] Tortora F., Cirillo M., Ferrara M., Belfiore M.P., Carella C., Caranci F., Cirillo S. (2013). Disease activity in Graves’ ophthalmopathy: Diagnosis with orbital MR imaging and correlation with clinical score. Neuroradiol. J..

[16] Chung W.J., Chung H.W., Shin M.J., Lee S.H., Lee M.H., Lee J.S., Kim M.J., Lee W.K. (2012). MRI to differentiate benign from malignant soft-tissue tumours of the extremities: A simplified systematic imaging approach using depth, size and heterogeneity of signal intensity. Br. J. Radiol..

[17] Fatima Z., Ichikawa T., Ishigame K., Motosugi U., Waqar A.B., Hori M., Iijima H., Araki T. (2014). Orbital masses: The usefulness of diffusion-weighted imaging in lesion categorization. Clin. Neuroradiol..

[18] Razek A.A., Elkhamary S., Mousa A. (2011). Differentiation between benign and malignant orbital tumors at 3-T diffusion MR-imaging. Neuroradiology.

[19] Pu J.J., Lo A.W.I., Wong M.C.M., Choi W.S., Ho G., Yang W.F., Su Y.X. (2024). A quantitative comparison of bone resection margin distances in virtual surgical planning versus histopathology: A prospective study. Int. J. Surg..

[20] Li R., Liu K., Hu Q., Shen J., Zuo D., Wang H., Zhu X., Sun W. (2025). NIR-II AIEgens nanosystem for fluorescence and chemiluminescence synergistic imaging-guided precise resection in osteosarcoma surgery. Aggregate.

[21] Polachova M., Netukova M., Benada O., Kucera T., Kolin V., Baxant A.-D., Sirolova Z., Studeny P. (2023). The new future perspective in corneal tissue utilisation–methods of preparation and preservation. BMC Ophthalmol..

[22] Wang S., Chen A., Yang L., Cai L., Xie Y., Fujimoto J., Gazdar A., Xiao G. (2018). Comprehensive analysis of lung cancer pathology images to discover tumor shape and boundary features that predict survival outcome. Sci. Rep..

[23] Shao Y., Chen Y., Chen S., Wei R. (2023). Radiomics analysis of T1WI and T2WI magnetic resonance images to differentiate between IgG4-related ophthalmic disease and orbital MALT lymphoma. BMC Ophthalmol..

[24] Sigmund E.E., Cho G.Y., Kim S., Finn M., Moccaldi M., Jensen J.H., Sodickson D.K., Goldberg J.D., Formenti S., Moy L. (2011). Intravoxel incoherent motion imaging of tumor microenvironment in locally advanced breast cancer. Magn. Reson. Med..

[25] Bhattacharjee R., Gupta M., Singh T., Sharma S., Khanna G., Parvaze S.P., Patir R., Vaishya S., Ahlawat S., Singh A. (2022). Role of intra-tumoral vasculature imaging features on susceptibility weighted imaging in differentiating primary central nervous system lymphoma from glioblastoma: A multiparametric comparison with pathological validation. Neuroradiology.

[26] Zhang L., Xie G., Zhang Y., Li J., Tang W., Yang L., Li K. (2024). A CT-based machine learning model for using clinical-radiomics to predict malignant cerebral edema after stroke: A two-center study. Front. Neurosci..

[27] Heyn C., Bishop J., Moody A.R., Kang T., Wong E., Howard P., Maralani P., Symons S., MacIntosh B.J., Keith J. (2025). Gadolinium-Enhanced T2 FLAIR Is an Imaging Biomarker of Radiation Necrosis and Tumor Progression in Patients with Brain Metastases. AJNR. Am. J. Neuroradiol..

[28] Tang W., Hei Y., Xiao L. (2013). Recurrent orbital space-occupying lesions: A clinicopathologic study of 253 cases. Chin. J. Cancer Res..

[29] Aerts H.J., Velazquez E.R., Leijenaar R.T., Parmar C., Grossmann P., Carvalho S., Bussink J., Monshouwer R., Haibe-Kains B., Rietveld D. (2014). Decoding tumour phenotype by noninvasive imaging using a quantitative radiomics approach. Nat. Commun..

[30] Liao P.Y., Dong Z.Y., Huang C.T., Tang X.R., Liu G.D., Liu Z., Wu D.H. (2020). Development and Validation of a Prognostic Nomogram Based on Residual Tumor in Patients With Nondisseminated Nasopharyngeal Carcinoma. Technol. Cancer Res. Treat..

[31] Yan J., Zhou S., Li Y. (2012). Benign orbital tumors with bone destruction in children. PLoS ONE.

[32] Shinder R., Al-Zubidi N., Esmaeli B. (2011). Survey of orbital tumors at a comprehensive cancer center in the United States. Head Neck.

[33] Graef S., DeAngelis D., Gupta A.A., Wan M.J. (2024). Ocular manifestations and long-term complications of rhabdomyosarcoma in children. Eye.

[34] Osaki T.H., Jakobiec F.A., Mendoza P.R., Lee Y., Fay A.M. (2013). Immunohistochemical investigations of orbital infantile hemangiomas and adult encapsulated cavernous venous lesions (malformation versus hemangioma). Ophthalmic Plast. Reconstr. Surg..

[35] Dufner Krieger S., Hidalgo Ramos R.A., Secades D., Hong I., Ortiz M. (2025). Rhabdomyosarcoma: A Case Report and Comprehensive Literature Review. Cureus.

[36] Clarós P., Choffor-Nchinda E., Lopez-Fortuny M., Claros A., Quintana S. (2019). Orbital cavernous haemangioma; profile and outcome of 76 patients managed surgically. Acta Oto-Laryngol..

[37] Lope L.A., Hutcheson K.A., Khademian Z.P. (2010). Magnetic resonance imaging in the analysis of pediatric orbital tumors: Utility of diffusion-weighted imaging. J. Am. Assoc. Pediatr. Ophthalmol. Strabismus.

[38] Naran S., MacIsaac Z., Katzel E., Bykowski M., Shakir S., Goldstein J., Pollack I.M., Losee J.E. (2016). Pediatric Craniofacial Fractures: Trajectories and Ramifications. J. Craniofacial Surg..

[39] Saito J.M., Yan Y., Evashwick T.W., Warner B.W., Tarr P.I. (2013). Use and accuracy of diagnostic imaging by hospital type in pediatric appendicitis. Pediatrics.

[40] Firriolo J.M., Ontiveros N.C., Pike C.M., Taghinia A.H., Rogers-Vizena C.R., Ganor O., Greene A.K., Meara J.G., Labow B.I. (2017). Pediatric Orbital Floor Fractures: Clinical and Radiological Predictors of Tissue Entrapment and the Effect of Operative Timing on Ocular Outcomes. J. Craniofacial Surg..

[41] Weaver J.M., DiPiero M., Rodrigues P.G., Cordash H., Davidson R.J., Planalp E.M., Dean D.C. (2023). Automated motion artifact detection in early pediatric diffusion MRI using a convolutional neural network. Imaging Neurosci..

[42] Mayer S.L., Brajcich M.R., Juste L., Hsu J.Y., Yehya N. (2024). Racial and Ethnic Disparity in Approach for Pediatric Intensive Care Unit Research Participation. JAMA Netw. Open.

[43] Rees C.A., Monuteaux M.C., Herdell V., Fleegler E.W., Bourgeois F.T. (2021). Correlation Between National Institutes of Health Funding for Pediatric Research and Pediatric Disease Burden in the US. JAMA Pediatr..

[44] Li R., Tian S., Zhu Y., Zhu W., Wang S. (2020). Management of orbital invasion in sinonasal squamous cell carcinoma: 15 years’ experience. Int. Forum Allergy Rhinol..

[45] Spinos D., Kalamatianos T., Terzakis D., Piagkou M., Georgalas C. (2021). Treatment strategies for inverted papillomas with intracranial or intraorbital involvement. J. Laryngol. Otol..

[46] Wang J., Ford J., Esmaeli B., Langer P., Esmaili N., Griepentrog G.J., Couch S.M., Nguyen J., Gold K.G., Duerksen K. (2021). Inverted Papilloma of the Orbit and Nasolacrimal System. Ophthalmic Plast. Reconstr. Surg..

[47] de Keizer R.O., de Wolff-Rouendaal D., de Keizer R.J. (2015). Silent squamous cell carcinoma invading the orbit following the course of the zygomaticotemporal nerve. Orbit.

[48] Parmar C., Grossmann P., Bussink J., Lambin P., Aerts H.J.W.L. (2015). Machine Learning methods for Quantitative Radiomic Biomarkers. Sci. Rep..

[49] Zwanenburg A., Vallières M., Abdalah M.A., Aerts H.J.W.L., Andrearczyk V., Apte A., Ashrafinia S., Bakas S., Beukinga R.J., Boellaard R. (2020). The Image Biomarker Standardization Initiative: Standardized Quantitative Radiomics for High-Throughput Image-based Phenotyping. Radiology.

[50] Ganti A., Tajudeen B.A., Plitt M.A., Rossi I., Gattuso P., Batra P.S. (2018). Discordance in Preoperative and Postoperative Histopathology of Sinonasal Tumors. Am. J. Rhinol. Allergy.

[51] Xia Z., Lin N., Chen W., Qi M., Sha Y. (2024). Multiparametric MRI-based radiomics nomogram for predicting malignant transformation of sinonasal inverted papilloma. Clin. Radiol..

[52] Yan Y., Liu Y., Tao J., Li Z., Qu X., Guo J., Xian J. (2022). Preoperative Prediction of Malignant Transformation of Sinonasal Inverted Papilloma Using MR Radiomics. Front. Oncol..

[53] Açar G., Büyükmumcu M., Güler İ. (2019). Computed tomography based analysis of the lamina papyracea variations and morphology of the orbit concerning endoscopic surgical approaches. Braz. J. Otorhinolaryngol..

[54] Gu J., Yu Q., Li Q., Peng J., Lv F., Gong B., Zhang X. (2022). MRI radiomics-based machine learning model integrated with clinic-radiological features for preoperative differentiation of sinonasal inverted papilloma and malignant sinonasal tumors. Front. Oncol..

[55] Wang L., Peng J., Wen B., Zhai Z., Yuan S., Zhang Y., Ii L., Li W., Ding Y., Wang Y. (2025). Contrast-Enhanced Computed Tomography-Based Machine Learning Radiomics Predicts IDH1 Expression and Clinical Prognosis in Head and Neck Squamous Cell Carcinoma. Acad. Radiol..

[56] Li G., Liu H., Pan Z., Cheng L., Dai J. (2025). Predicting craniofacial fibrous dysplasia growth status: An exploratory study of a hybrid radiomics and deep learning model based on computed tomography images. Oral Surg. Oral Med. Oral Pathol. Oral Radiol..

[57] Qi M., Sha Y., Zhang D., Ren J. (2023). An MRI-based radiomics nomogram for detecting cervical esophagus invasion in hypopharyngeal squamous cell carcinoma. Cancer Imaging Off. Publ. Int. Cancer Imaging Soc..

[58] He S., Zhao Y., Shi L., Yang X., Wang X., Luo Y., Wang M., Zhang X., Li X., Yu D. (2024). Utilizing radiomics for differential diagnosis of inverted papilloma and chronic rhinosinusitis with polyps based on unenhanced CT scans. Sci. Rep..

[59] Xu J., Qin L., Wang D. (2022). Analysis of the Lamina Papyracea Dehiscence Based on Computed Tomography Findings. J. Craniofacial Surg..

[60] Mirabella T., MacArthur J.W., Cheng D., Ozaki C.K., Woo Y.J., Yang M., Chen C.S. (2020). Author Correction: 3D-printed vascular networks direct therapeutic angiogenesis in ischaemia. Nat. Biomed. Eng..

[61] Costa P.F., Albers H.J., Linssen J.E.A., Middelkamp H.H.T., van der Hout L., Passier R., van den Berg A., Malda J., van der Meer A.D. (2017). Mimicking arterial thrombosis in a 3D-printed microfluidic in vitro vascular model based on computed tomography angiography data. Lab Chip.

[62] Hanna C., Danis D., Kimia R., Merkouris H., Steinwald P., O’Leary M., Tracy J. (2025). Donor site morbidity of upper extremity flaps in head and neck reconstruction. Am. J. Otolaryngol..

[63] Ibelli T.J., Sayegh F., Kagen A.C., Henderson P.W. (2022). FlapMap Visual Language System for Vascular Imaging Prior to Microvascular Free Tissue Transfer. Plastic and reconstructive surgery. Glob. Open.

[64] Rahman A.S., Wang V.J., Paderno A., Bedi N., Montalbaron M., Damrose E., Chen M.M., Finegersh A., Lee J.J., Holsinger F.C. (2025). Improved Visualization of Oropharyngeal Tumors Using Simple Maneuvers During Flexible Endoscopy. Ann. Otol. Rhinol. Laryngol..

[65] Li C., Cai Y., Wang W., Sun Y., Li G., Dimachkieh A.L., Tian W., Sun R. (2019). Combined application of virtual surgery and 3D printing technology in postoperative reconstruction of head and neck cancers. BMC Surg..

[66] Panesar S.S., Magnetta M., Mukherjee D., Abhinav K., Branstetter B.F., Gardner P.A., Iv M., Fernandez-Miranda J.C. (2019). Patient-specific 3-dimensionally printed models for neurosurgical planning and education. Neurosurg. Focus.

[67] Ngaage L.M., Hamed R., Oni G., Di Pace B., Ghorra D.T., Koo BMedSci B.C., Malata C.M. (2019). The Role of CT Angiography in Assessing Deep Inferior Epigastric Perforator Flap Patency in Patients With Pre-existing Abdominal Scars. J. Surg. Res..

[68] Cao Z.M., Du W., Qing L.M., Zhou Z.B., Wu P.F., Yu F., Pan D., Xiao Y.B., Pang X.Y., Liu R. (2019). Reconstructive surgery for foot and ankle defects in pediatric patients: Comparison between anterolateral thigh perforator flaps and deep inferior epigastric perforator flaps. Injury.

[69] Zhang X., Cao Y.Z., Mu X.H., Sun Y., Schmidt M., Forman C., Speier P., Lu S.S., Hong X.N. (2020). Highly accelerated compressed sensing time-of-flight magnetic resonance angiography may be reliable for diagnosing head and neck arterial steno-occlusive disease: A comparative study with digital subtraction angiography. Eur. Radiol..

[70] Romano A., Tavanti F., Rossi Espagnet M.C., Terenzi V., Cassoni A., Suma G., Boellis A., Pierallini A., Valentini V., Bozzao A. (2015). The role of time-resolved imaging of contrast kinetics (TRICKS) magnetic resonance angiography (MRA) in the evaluation of head-neck vascular anomalies: A preliminary experience. Dento Maxillo Facial Radiol..

[71] de Geer A.F., Mulder I., Ter Beek L.C., Te Boekhorst A.S., Plakké B.I., Karssemakers L.H.E., Dirven R., Lohuis P.J.F.M., Ruers T.J.M., Siepel F.J. (2025). Perforator mapping for head and neck reconstructive surgery: A novel personalized approach using magnetic resonance angiography based 3D models and 3D-printing. Front. Oncol..

[72] Zhou J., Zuo E., Ding Y., Wu J., Jin Y., Chen X. (2024). Effect of Preoperative Neck Radiotherapy on the Reconstruction of Head and Neck Defects With the Supraclavicular Artery Island Flap. Ear Nose Throat J..

[73] Munisso M.C., Liu C., Yamamoto G., Kosaka T., Tsuge I., Saito S., Morimoto N. (2024). PreFlap: From Photoacoustic Tomography Images to Vascular Mapping Sheets for Improved Preoperative Flap Evaluation. IEEE Trans. Bio-Med. Eng..

[74] Su T., Pirgousis P., Fernandes R. (2013). Versatility of supraclavicular artery island flap in head and neck reconstruction of vessel-depleted and difficult necks. J. Oral Maxillofac. Surg. Off. J. Am. Assoc. Oral Maxillofac. Surg..

[75] Ota Y., Moore A.G., Spector M.E., Casper K., Stucken C., Malloy K., Lobo R., Baba A., Srinivasan A. (2022). Prediction of Wound Failure in Patients with Head and Neck Cancer Treated with Free Flap Reconstruction: Utility of CT Perfusion and MR Perfusion in the Early Postoperative Period. AJNR. Am. J. Neuroradiol..

[76] Razavi C.R., Hostetter J., Shukla A., Cheng Z., Aygun N., Boahene K., Byrne P.J., Richmon J., Quon H., Desai S.C. (2022). Predictors of Free Flap Volume Loss in Nonosseous Reconstruction of Head and Neck Oncologic Defects. Ear Nose Throat J..

[77] Kim M.J., Oh T.S. (2020). A nasolabial fold reset technique for enhancing midface lifts in facial reanimation: Three-dimensional volumetric analysis. J. Cranio-Maxillo-Facial Surg. Off. Publ. Eur. Assoc. Cranio-Maxillo-Facial Surg..

[78] Hennocq Q., Caruhel J.B., Benassarou M., Bouaoud J., Chaine A., Girod A., Graillon N., Testelin S., Amor-Sahli M., Foy J.P. (2025). Predictive Factors of Free Flap Volume Evolution in Head and Neck Reconstruction. Otolaryngol. Neck Surg..

[79] Kohler L.H., Köhler H., Kohler S., Langer S., Nuwayhid R., Gockel I., Spindler N., Osterhoff G. (2021). Hyperspectral Imaging (HSI) as a new diagnostic tool in free flap monitoring for soft tissue reconstruction: A proof of concept study. BMC Surg..

[80] Thiem D.G.E., Römer P., Blatt S., Al-Nawas B., Kämmerer P.W. (2021). New Approach to the Old Challenge of Free Flap Monitoring-Hyperspectral Imaging Outperforms Clinical Assessment by Earlier Detection of Perfusion Failure. J. Pers. Med..

[81] Felicio-Briegel A., Linek M., Sroka R., Rühm A., Freymüller C., Stocker M., Baumeister P., Reichel C., Volgger V. (2024). Hyperspectral imaging for monitoring of free flaps of the oral cavity: A feasibility study. Lasers Surg. Med..

[82] Wong O.L., Yuan J., Zhou Y., Yu S.K., Cheung K.Y. (2021). Longitudinal acquisition repeatability of MRI radiomics features: An ACR MRI phantom study on two MRI scanners using a 3D T1W TSE sequence. Med. Phys..

[83] Dupuis A., Chen Y., Hansen M., Chow K., Sun J.E.P., Badve C., Ma D., Griswold M.A., Boyacioglu R. (2024). Quantifying 3D MR fingerprinting (3D-MRF) reproducibility across subjects, sessions, and scanners automatically using MNI atlases. Magn. Reson. Med..

[84] Kim B., Yoon D.Y., Seo Y.L., Park M.W., Kwon K.H., Rho Y.S., Chung C.H. (2017). Value of the Post-Operative CT in Predicting Delayed Flap Failures Following Head and Neck Cancer Surgery. Korean J. Radiol..

[85] Simianu V.V., Morris A.M., Varghese T.K., Porter M.P., Henderson J.A., Buchwald D.S., Flum D.R., Javid S.H. (2016). Evaluating disparities in inpatient surgical cancer care among American Indian/Alaska Native patients. Am. J. Surg..

[86] Abella M.K.I.L., Lee A.Y., Kitamura R.K., Ahn H.J., Woo R.K. (2023). Disparities and Risk Factors for Surgical Complication in American Indians and Native Hawaiians. J. Surg. Res..

[87] Hsia R., Shen Y.C. (2011). Possible geographical barriers to trauma center access for vulnerable patients in the United States: An analysis of urban and rural communities. Arch. Surg..

[88] Dulaney A., Virostko J. (2024). Disparities in the Demographic Composition of The Cancer Imaging Archive. Radiology. Imaging Cancer.

[89] Alabi R.O., Elmusrati M., Leivo I., Almangush A., Mäkitie A.A. (2024). Artificial Intelligence-Driven Radiomics in Head and Neck Cancer: Current Status and Future Prospects. Int. J. Med. Inform..

[90] Tejani A.S., Ng Y.S., Xi Y., Rayan J.C. (2024). Understanding and Mitigating Bias in Imaging Artificial Intelligence. RadioGraphics.

[91] Yi P.H., Bachina P., Bharti B., Garin S.P., Kanhere A., Kulkarni P., Li D., Parekh V.S., Santomartino S.M., Moy L. (2025). Pitfalls and Best Practices in Evaluation of AI Algorithmic Biases in Radiology. Radiology.

[92] Patel R., Provenzano D., Wallington S., Loew M., Rao Y., Goyal S. (2023). Racial/ethnic reporting differences in cancer literature regarding machine learning vs. a radiologist: A systematic review and meta-analysis. J. Med. Artif. Intell..

[93] Rao A.A., Naheedy J.H., Chen J.Y., Robbins S.L., Ramkumar H.L. (2013). A clinical update and radiologic review of pediatric orbital and ocular tumors. J. Oncol..

[94] Hua S.B.Z., Heller N., He P., Towbin A.J., Chen I.Y., Lu A.X., Erdman L. (2025). Lack of Children in Public Medical Imaging Data Points to Growing Age Bias in Biomedical AI. medRxiv.

[95] Zwanenburg A., Leger S., Vallières M., Löck S. (2016). Image Biomarker Standardisation Initiative-Feature Definitions. arXiv.

[96] Shao Y., Ma J.M., Huang X.M. (2025). Guidelines for standard operation of imaging modalities in orbital diseases (2024). Int. J. Ophthalmol..

[97] Paquier Z., Chao S., Bregni G., Sánchez A., Guiot T., Dhont J., Gulyban A., Levillain H., Sclafani F., Reynaert N. (2022). Pre-trial quality assurance of diffusion-weighted MRI for radiomic analysis and the role of harmonisation. Phys. Medica.

[98] Selim M., Zhang J., Fei B., Zhang G., Chen J. (2021). CT Image Harmonization for Enhancing Radiomics Studies. Proceedings of the 2021 IEEE International Conference on Bioinformatics and Biomedicine (BIBM).

[99] Orlhac F., Boughdad S., Philippe C., Stalla-Bourdillon H., Nioche C., Champion L., Soussan M., Frouin F., Frouin V., Buvat I. (2018). A Postreconstruction Harmonization Method for Multicenter Radiomic Studies in PET. J. Nucl. Med..

[100] Orlhac F., Frouin F., Nioche C., Ayache N., Buvat I. (2019). Validation of A Method to Compensate Multicenter Effects Affecting CT Radiomics. Radiology.

[101] Yang Y., Zhang H., Gichoya J.W., Katabi D., Ghassemi M. (2024). The limits of fair medical imaging AI in real-world generalization. Nat. Med..

[102] Chetan M., Gleeson F. (2020). Radiomics in predicting treatment response in non-small-cell lung cancer: Current status, challenges and future perspectives. Eur. Radiol..

[103] Cobo M., Fernández-Miranda M., Bastarrika G., Iglesias L. (2023). Enhancing radiomics and Deep Learning systems through the standardization of medical imaging workflows. Sci. Data.

[104] Kumar V., Gu Y., Basu S., Berglund A., Eschrich S.A., Schabath M.B., Forster K., Aerts H.J., Dekker A., Fenstermacher D. (2012). Radiomics: The process and the challenges. Magn. Reson. Imaging.

[105] Lefebvre T., Ueno Y., Dohan A., Chatterjee A., Vallières M., Winter-Reinhold E., Saif S., Levesque I., Zeng X., Forghani R. (2022). Development and Validation of Multiparametric MRI-based Radiomics Models for Preoperative Risk Stratification of Endometrial Cancer. Radiology.

[106] Forouzannezhad P., Maes D., Hippe D., Thammasorn P., Iranzad R., Han J., Duan C., Liu X., Wang S., Chaovalitwongse W. (2022). Multitask Learning Radiomics on Longitudinal Imaging to Predict Survival Outcomes following Risk-Adaptive Chemoradiation for Non-Small Cell Lung Cancer. Cancers.

[107] Starke S., Zwanenburg A., Leger K., Zöphel K., Kotzerke J., Krause M., Baumann M., Troost E., Löck S. (2023). Longitudinal and Multimodal Radiomics Models for Head and Neck Cancer Outcome Prediction. Cancers.

[108] Von Ende E., Ryan S., Crain M., Makary M. (2023). Artificial Intelligence, Augmented Reality, and Virtual Reality Advances and Applications in Interventional Radiology. Diagnostics.

[109] Mangano F., Admakin O., Lerner H., Mangano C. (2023). Artificial Intelligence and Augmented Reality for Guided Implant Surgery Planning: A Proof of Concept. J. Dent..

[110] Chan H., Haerle S., Daly M., Zheng J., Philp L., Ferrari M., Douglas C., Irish J. (2021). An integrated augmented reality surgical navigation platform using multi-modality imaging for guidance. PLoS ONE.

[111] Mazurowski M.A., Buda M., Saha A., Bashir M.R. (2019). Deep learning in radiology: An overview of the concepts and a survey of the state of the art with focus on MRI. J. Magn. Reson. Imaging.

[112] Chang L., Liu J., Zhu J., Guo S., Wang Y., Zhou Z., Wei X. (2025). Advancing precision medicine: The transformative role of artificial intelligence in immunogenomics, radiomics, and pathomics for biomarker discovery and immunotherapy optimization. Cancer Biol. Med..

[113] Liu Z., Wang S., Dong D., Wei J., Fang C., Zhou X., Sun K., Li L., Li B., Wang M. (2019). The Applications of Radiomics in Precision Diagnosis and Treatment of Oncology: Opportunities and Challenges. Theranostics.

[114] Lyu W., Gong J., Zhu L., Xu T., Huang S., Shen C., Wang C., He X., Ying H., Hu C. (2024). MR radiomics unveils neoadjuvant chemo-responsiveness with insights into selective treatment de-intensification in HPV-positive oropharyngeal carcinoma. Oral Oncol..

[115] Avanzo M., Wei L., Stancanello J., Vallières M., Rao A., Morin O., Mattonen S.A., El Naqa I. (2020). Machine and deep learning methods for radiomics. Med. Phys..

[116] Pinker K., Chin J., Melsaether A., Morris E., Moy L. (2018). Precision Medicine and Radiogenomics in Breast Cancer: New Approaches toward Diagnosis and Treatment. Radiology.

[117] Chiesa S., Russo R., Bartoli B., Palumbo I., Sabatino G., Cannatà M., Gigli R., Longo S., Tran H., Boldrini L. (2023). MRI-derived radiomics to guide post-operative management of glioblastoma: Implication for personalized radiation treatment volume delineation. Front. Med..

[118] Hacking S., Yakirevich E., Wang Y. (2022). From Immunohistochemistry to New Digital Ecosystems: A State-of-the-Art Biomarker Review for Precision Breast Cancer Medicine. Cancers.

[119] Boehm K., Khosravi P., Vanguri R., Gao J., Shah S. (2021). Harnessing multimodal data integration to advance precision oncology. Nat. Rev. Cancer.

